# “It’s on the ‘nice to have’ pile”: Potential principles to improve the implementation of socially inclusive Green Infrastructure

**DOI:** 10.1007/s13280-020-01372-2

**Published:** 2020-09-30

**Authors:** Dan Fisher, Kirsty Blackstock, Katherine Irvine

**Affiliations:** The James Hutton Institute, Craigiebuckler, Aberdeen, AB15 8QH Scotland, UK

**Keywords:** Green Infrastructure, Implementation, Nature-based solutions, Planning, Social principles, Urban greening

## Abstract

Green Infrastructure (GI) research tends to focus on the need for GI to enhance ecological processes, its potential to provide health and economic benefits, and on the barriers preventing its uptake. Yet there has been inadequate focus on the social aspects of GI. In the United Kingdom (UK) the need for GI is well established, such that policymakers and planners are now turning to the question of how GI should be implemented. Drawing on a mixed method research approach centring on practitioner experience, this paper identifies potential social principles that underpin GI and questions the extent to which these are being implemented in the UK. Results highlight the hitherto unexplored complexities of GI maintenance, the effects of austerity politics, and the role of local-level power dynamics on the implementation of GI. Findings have implications for international literature on GI as well as nature-based solutions more broadly.

**Electronic supplementary material:**

The online version of this article (10.1007/s13280-020-01372-2) contains supplementary material, which is available to authorized users.

## Introduction

‘Nature-based solutions’ (NBS) is often understood as an umbrella term to collate various existing concepts and approaches to enhance nature and its benefits for people (Albert et al. 2019). Although the relationship between Green Infrastructure (GI) and NBS is not yet well-defined, GI has frequently been understood as a form of NBS (Nesshöver et al. 2017; Keestra et al. 2018). We follow Pauleit et al.’s (2017, p. 29) proposal that GI be understood as providing “strategic guidance for the integration of NBS into developing multifunctional green space networks at various scales.” This strategic guidance is necessary as sustainable NBS solutions that ensure the monitoring of environmental targets, equitable distribution of benefits and risks, long-term financial investments and the development of appropriate institutional arrangements are still being designed (Nesshöver et al. 2017; see also Haase et al. 2017). As will be established in this paper, however, such strategic guidance being offered by GI is, in practice, both unclear and challenging to implement. This lack of clarity therefore poses problems for both GI as well as NBS more broadly. Focusing predominantly on the social aspects of GI, this paper makes an original contribution to the literature on GI and NBS by putting the viewpoints of stakeholders and practitioners at the centre of the debate concerning the social principles of GI and the challenges surrounding their implementation.

There have been long-standing discussions amongst academics, practitioners and policymakers as to what the term GI means. While it is generally accepted that GI refers to connected green (and blue) spaces that are strategically designed and managed to provide multiple functions and human benefits, many definitions exist (see Benedict and McMahon 2002; Kambites and Owen 2006). Agreeing on what GI should look like and how it should be implemented in practice has, therefore, largely alluded academics and practitioners alike. Due to the malleability of the concept (Lennon 2015), GI has become what Wright (2011, p. 1004) refers to as a ‘contested topic’; where, despite broadly accepted definitions, “different interests attach different environmental, social and economic meanings to it.”

Despite variations in its meaning, GI has now become an adopted concept at local, regional and international scales. At the European scale, for example, the European Union (EU) has advocated for the use and integration of GI into EU policy areas (European Commission 2016). In the United Kingdom (UK), GI has been inserted into the English National Planning Policy Framework (DEFRA 2018). In Scotland, the Scottish Planning Policy states that the planning system should consider “green infrastructure as an integral element of places from the outset of the planning process” (Scottish Government 2014, p. 50) and the Welsh planning policy requires planning authorities to develop robust approaches to enhancing biodiversity and increasing wellbeing through Green Infrastructure Assessments (Welsh Government 2018).

This widespread referencing of GI in policy and planning has led Jerome et al. (2019, p. 174) to state that the “advocacy argument [for GI] has largely been won.” Yet despite these efforts, the implementation of GI has been surprisingly slow (Matthews et al. 2015). Worse, as Jerome et al. (2019) lament, examples of high-quality GI are still the exception rather than the norm. This is unsurprising given the existing literature on challenges to GI implementation (O’Donnell et al. 2017). Various reasons have been put forward for these implementation challenges including: (1) frequent conflation of GI with traditional green spaces; (2) the continued silo-based approach to the policy issues to which GI could respond; (3) highly-variable local development plans that de-value GI in the planning process; (4) lack of consideration of long-term stewardship of GI; and (5) uncertainty as to what makes GI successful (see Scott et al. 2013; Matthews et al. 2015; Hislop et al. 2019; Jerome et al. 2017, 2019). It is the latter issue that forms the starting point of this paper.

Given the malleability of the concept, it is unsurprising that there is uncertainty concerning how to assess GI. While considerable evidence suggests that GI can provide multiple social and environmental benefits (Kambites and Owen 2006), does the provision of these benefits directly equate to achieving socio-economic inclusion? New York’s High Line is the most evident example of GI providing social and environmental benefits to the city, while simultaneously facing much criticism following its negative impacts on peoples’ sense of place and eco-gentrification effects (Patrick 2014; Lang and Rothenberg 2017). There are many such examples of GI increasing social stratification rather than serving the local community (Curran and Hamilton 2012; Wolch et al. 2014; Meerow and Newell 2017; Anguelovski et al. 2018; Rigolon and Németh 2018). Moreover, Finewood et al. (2019) demonstrate how GI can become depoliticised through its eventual transformation and adoption as primarily a *technical* solution to the problem of stormwater drainage—resulting in the drowning out of community actors’ voices previously engaged in the planning process. It is important, therefore, to explicitly address the social, cultural and political dimensions of GI throughout the project cycle.

Using the UK as an example, this paper is directed by three questions: (1) can a set of guiding social principles be adopted for GI? (2) What challenges do practitioners in the UK experience in their attempts to implement such principles? (3) Do the principles and their challenges vary across the project cycle? In this respect we build on the attempts of Roe and Mell (2013), Haase et al. (2017) and Jerome et al. (2019) to define a set of guiding principles relevant to GI, which should prove more valuable than attempting to provide another definition of GI (Mell 2013). In the following section we set out the research methods that were employed in order to ascertain the views of practitioners in the UK. We then present our findings and subsequently discuss the implications of our work in the remainder of the paper.

## Materials and methods

To unpack GI’s social principles and their implementation, a literature review was first conducted. This review traced the evolution of the GI in academia (e.g. Benedict and McMahon 2002, 2006; Kambites and Owen 2006; Tzoulas et al. 2007) and analysed subsequent review articles (e.g. Wright 2011; Hansen and Pauleit 2014; Lennon 2015) and those focused on the challenge of providing socially inclusive GI (e.g. Anguelovski 2016; Haase et al. 2017; Anguelovski et al. 2018). Good practice guidance and explanatory documents in relevant grey literature were also reviewed (e.g. Natural England 2009; Scottish Government 2014; UKGBC 2018). Through this review, a list of social principles was identified. To develop a sense of practitioners’ use of the term GI, as well as the challenges being encountered in the UK context, one of the authors (DF) attended two practitioner-focused UK-based conferences (the Town and Country Planning Association & Green Infrastructure Partnership Conference: Achieving Better Green Infrastructure held in London 2019, and the Future Planning: Designing Places in a Climate Emergency conference held in Glasgow 2019).

To form our research sample, participants with explicit involvement in GI were sought from across the UK. Researchers used the conference attendance lists to generate an initial set of possible contacts, through which we reached out to 31 potential participants by email and follow-up phone calls.^1^ We also employed a snowball approach, asking research participants to suggest other practitioners they thought would be key for the purposes of this study (22 contacts). Researchers also targeted additional potential participants identified through online searches to increase geographic variation and employer type; this generated a further 18 contacts. Twenty practitioners agreed to take part in the research project; four subsequently cancelled and one could not complete the second stage. These cancellations were in part due to practitioners’ busy schedules as well as the outbreak of COVID-19. Participants were predominantly located in Scotland (8) and England (6), with one individual from Wales and Northern Ireland (Table 1). Despite the small sample, it was nevertheless sufficient to produce data saturation in the interviews, with researchers frequently hearing the same concerns and viewpoints from participants (Grady 1998). Participants were given a participant number (PN) to ensure anonymisation. Research ethics was obtained from the James Hutton Institute’s Research Ethics Committee (reference 186/2019).

**Table 1:** The list of participants in the study and notes their employer type

Participant number (PN)	Employer type	Employment role
1^a^	Developer	Executive Chair
2^a^	Consulting Landscape Architect	Director
3	Environmental Agency	Senior Planning Officer
4	Environmental Consultancy	Associate
5	Local Government	Senior Planning Officer
6	Environmental NGO	Director
7	Local Government	Planning Officer
8	Public Partnership	Programme Manager
9	Local Government	Biodiversity Officer
10	Local Government	Principal Design Officer
11	Environmental Agency	Placemaking Officer
12	Trade Body	Policy Lead
13	Environmental Consultancy	Director
14	Local Government	Principal Landscape Architect
15	Environmental Consultancy	Senior Landscape Architect
16^b^	Local Government	Senior Planning Officer/Planning Policy Officer

^a^Participants answered the pilot survey, which did not include the ‘ability to implement’ section of the survey

^b^Participants could not complete the interview due to time constraints

Researchers devised a mixed methods approach, combining survey data with qualitative interviews. Mixed methods are useful for further exploring quantitative results or generalising qualitative findings (Creswell and Piano Clark 2018). In this case, we employed the survey to ascertain practitioners’ general views concerning the proposed social principles and the extent to which they are currently applied. The interviews were subsequently used to unpack the reasons behind their views concerning the principles, the challenges faced in implementing the principles and the solutions the practitioners apply or envisage.

The survey was built and distributed online using the Qualtrics XM platform (a blank version of the survey can be read in the electronic supplementary material). We summarised our initial list of social principles into a set of 14 principles to minimise participant burden and allow for meaningful engagement (Table 2). Principles were phrased in such a way as to minimise social desirability bias and provoke discussion in the interviews. Adapting the RIBA (2020) approach, we defined 4 stages of project implementation and the actors frequently involved in each stage (Table 3). The 14 principles and 4 stages formed the basis of a survey with three main sections. Participants were asked: (1) to what extent each principle should be applied to GI; (2) at which stage each principle should be applied; and (3) to what extent participants have felt able to apply each principle (see supporting materials). Except for the first principle, participants were able to define their own understanding of the spatial scale at which principles should be applied. During the interviews, most participants referred to GI at the urban scale or local scale, as opposed to at the landscape scale. Skip logic was applied to the survey; principles that participants either disagreed or strongly disagreed with in the first section did not reappear in the following two sections of the survey. As a result, some principles in sections two and three of the survey have lower response rates than others. Participants were additionally asked for their definition of GI and to answer several background questions (e.g. socio-demographic, role within their organisation). Descriptive statistics were run for survey results which were used to tailor questions for the interview with each participant.

**Table 2:** The summarised list of social principles adapted from the GI literature

Principle	Shorthand	References
1. Green Infrastructure should include small-scale interventions that evenly distribute access to nature for all residents.	1. Small-scale interventions	Breuste (2010), Ignatieva and Ahrne (2013), Lovell and Taylor (2013), Wolch et al. (2014)
2. Funding for Green Infrastructure should cover the full life-cycle of projects (i.e. including maintenance and monitoring costs).	2. Funding covers life-cycle	Kambites and Owen (2006), O’Donnell et al. (2017)
3. There should be regular checks or audits in place to ensure that Green Infrastructure projects comply with relevant policies and procedures.	3. Audits for policy compliance	Natural England (2009), UKGBC (2015, 2018)
4. The preferences of residents and stakeholder groups should be incorporated into Green Infrastructure projects, even if these limit other goals.	4. Stakeholder views incorporated	Benedict and McMahon (2002), Faehnle et al. (2014), Wilker et al. (2016), Haase et al. (2017), Anguelovski et al. (2018), Pauleit et al. (2019)
5. There should be national Green Infrastructure standards that are embedded within planning and social policy.	5. National GI standards	Natural England (2009), McLintock (2018)
6. There should be clear targets and responsibilities in the monitoring and maintenance of Green Infrastructure projects post-installation.	6. Maintenance strategy agreed	Natural England (2009), TCPA and Wildlife Trust (2012), UKGBC (2015)
7. Socio-economic trade-offs associated with Green Infrastructure need to be considered, and negative impacts minimised especially in areas of high inequality.	7. Consideration of trade-offs	Lin et al. (2015), Miller (2016), Haase et al. (2017), Rigolon and Németh (2018), Anguelovski et al. (2019)
8. Green Infrastructure should be in keeping with existing land uses and cultural contexts of an area, even if these are ‘industrial’.	8. Maintain cultural contexts	Curran and Hamilton (2012), Miller (2016), Jerome et al. (2019)
9. Private profit should not be prioritised over public interest when seeking funding from private actors for Green Infrastructure.	9. Public interest over profit	Curran and Hamilton (2012), Wolch et al. (2014), Haase et al. (2017)
10. Access for all users throughout the year should be included in Green Infrastructure.	10. Access for all users	CABE (2010), TCPA and Wildlife Trusts (2012), Greed (2015), Manley (2015), Jerome et al. (2019)
11. Evidence from completed projects should be used to revise Green Infrastructure goals and future projects.	11. Evidence for future	Bowen and Parry (2015), Hansen et al. (2017)
12. Green Infrastructure should help bring communities together.	12. Bring communities together	Burgess (2015), Haase et al. (2017), DEFRA (2018)
13. Green Infrastructure projects should be inclusive of minority and disadvantaged groups, working to ensure they benefit following installation.	13. Inclusive of minority groups	Benedict and McMahon (2002), Dunn (2010), Kabisch and Haase (2014), Hansen et al. (2017)
14. Green Infrastructure should enhance community resilience (i.e. the ability of a community to use locally-available resources and withstand adverse situations).	14. GI enhances resilience	Lennon and Scott (2014), Meerow and Newell (2017), Shokry et al. (2020)

**Table 3:** The stages of GI implementation as described to the participants in the survey

Stage 1: Policy and planning—refers to government policy-setting concerning Green Infrastructure, as well as steering of the GI concept by e.g. built environment organisations and consultancies Stage 2: Project concept and technical design—refers to the design and planning stage of specific GI projects that will be applied on a local scale by e.g. planners, architects, project management services in collaboration with local government Stage 3: Implementation and construction—is the stage during which plans are put into practice, often by developers and contractors that have not been involved in stages 1 and 2 Stage 4: Long-term management and monitoring—concerns the post-construction phase during which time GI needs to be maintained and used	

Following the completion of the survey, participants were interviewed either over the phone or using Cisco Webex to elucidate a more in-depth understanding of their interpretation of GI and the challenges they face in implementing it. In total 15 participants answered both the interview and the survey. Two surveys were conducted as pilot surveys and did not include the third section of the questionnaire (ability to apply principles). This third section was added to the survey following the interviews with those first being piloted, where overall responses indicated that there were significant problems implementing GI and also that answering the survey had taken the participants less time than anticipated. Both the qualitative data and survey results from the pilot participants have been included in the results, meaning responses concerning the applicability of principles are lower than the other sections. One participant could not conduct the interview after having completed the survey due to time constraints; their survey data were retained for analysis and included in the results.

Interviews lasted between 35 and 80 min and were structured around a participant’s survey responses—focusing on principles with which participants either disagreed in general or strongly agreed, and principles that they had either found easy to apply or not been able to apply. Interview questions were also targeted depending on participants’ specific roles and experiences. Interviews were transcribed and analysed through a qualitative thematic analysis. Themes were developed through an iterative process, rather than prescribed, to allow key themes to emerge from the data.

## Results

### Participant views concerning social GI principles

**Fig. 1:**
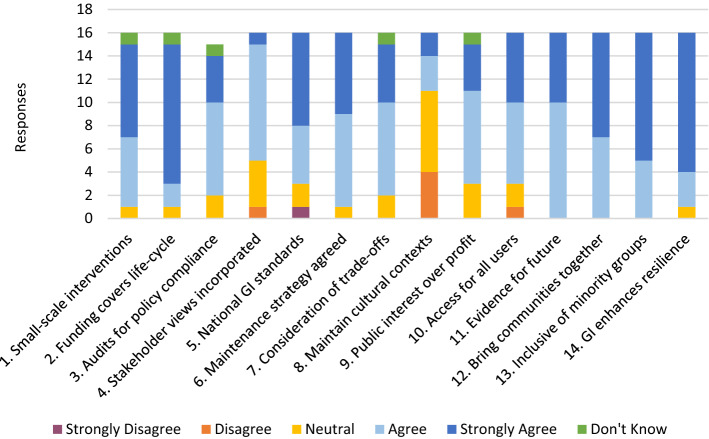
The extent to which participants agreed with the proposed social principles for GI

With few exceptions, there was a general broad agreement that the suggested social principles were applicable to GI with most participants indicating ‘agree’ or ‘strongly agree’ (Fig. 1). One participant indicated that they ‘did not know’ for Principles 1, 2, 3, 7, 9, explaining in the interview that in their view these principles lacked specificity or set up false choices. Participants expressed strongest agreement for principles which focus on equal access (principle 1), inclusivity (principle 13), community resilience (principle 14) and availability of funds throughout the project cycle (principle 2). Principles which focused on auditing (principle 3), setting standards (principle 5), accounting for socio-economic trade-offs (principle 6) and drawing on evidence from completed projects (principle 11) were considered applicable but not as strongly.

Although participants felt strongly that these were social principles that apply to GI, many also stated that these principles should apply to the planning system more generally. Following these social principles affords planners the opportunity to move away from what is seen as an overly adversarial planning system. Instead GI is:

[…] a good way to create the base for [the] discussion [between communities and the planning system] because it has such huge scope in terms of its capability to respond to the range of people’s needs […] it’s the means of creating a framework for the kind of places that not only the people want but that they actually need. (PN4)

There was a greater diversity of opinion regarding principles 4, 5, 8 and 10. Principle 4 (incorporation of stakeholders’ views) and principle 10 (access for all users) were each disagreed with once. Participants also felt that, although stakeholder preferences should be incorporated into designs, GI also involves educating people about the benefits of GI and that stakeholder preferences should not always be the foremost consideration. One individual strongly disagreed with the principle of national standards for GI (principle 5) stating that GI standards would be best set at a local level, given the already-congested landscape of planning standards, supported by clear national policy. Participants were largely in agreement that action needs to be taken to avoid developers ‘value-engineering’ projects. There was however little agreement as to how this could best be achieved, with some advocating a regulatory approach and others seeking a voluntary approach.

Principle 8, which focuses on maintenance of existing land uses and cultural contexts, generated the greatest diversity of opinion with four individuals disagreeing and many indicating neutrality. Due to the skip logic applied in the survey, responses were therefore lower concerning principle 8 in the second and third sections of the survey. Participants felt that, although GI should be largely in keeping with existing land use, this should also be context-dependent and not preclude the implementation of GI—especially where such land uses stand in the way of rewilding opportunities (e.g. industrial agricultural landscapes). In fact, participants opined that some principles lacked specificity and could not work for all types of GI. The need to define the local contexts in which these principles would be applied therefore led one participant to say:

[…] the idea of having social principles must be the right thing to do, but we have to be very wary about those principles […] Not all principles need apply in all circumstances. (PN9)

Therefore, despite some disagreement, there is general support for these principles amongst our participants.

### Ability to implement social GI principles

**Fig. 2:**
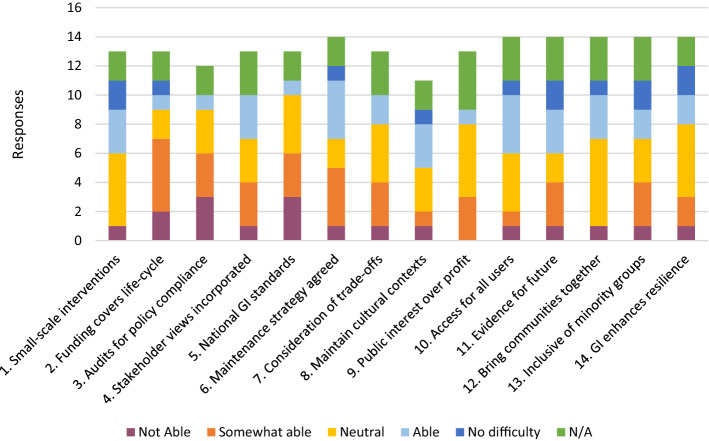
The extent to which participants felt they had been able to apply the suggested social principles in their recent work

Figure 2 illustrates whether participants felt able to apply the principles in their recent work. Most participants were able to answer this question, suggesting that these principles are being enacted to some extent already. This result might be slightly higher than the norm given that participants indicated a keen interest in improving the quality of GI implementation in the UK. In the interviews participants frequently made it clear that their experiences were not necessarily standard in the UK. For example,

[…] we’re conscious of how important it is to make sure that we try to have green infrastructure that is designed to be as easy and cost effective to maintain as possible […] But that’s not universal at all and that’s just purely down to lack of understanding [and a] lack of awareness at all levels really. (PN4)

Very few participants reported ‘no difficulty’ with the principles, which is to be expected given the literature on implementation challenges in GI and planning more generally. Two participants (PN7 and PN12) answered ‘not applicable’ for all questions in this section, stating in their interviews that they work at a strategic level and therefore work to promote these principles. One participant (PN13) was very positive in comparison to other participants, selecting ‘no difficulty’ for nine of the fourteen principles, which can be explained by the nature of their role as director of an environmental consultancy. One participant (PN14), by contrast, answered ‘not able at all’ for each principle. This negativity concerning the implementation of GI appears to be stronger for local government employees (*n* = 4), which derived from a broader frustration concerning the implementation of GI.

In many cases the distribution is even, with most participants selecting ‘neutral’ (principles 1, 7, 12 and 14). Principles 1, 6, 10, 11 and 12 had more positive responses than negative, suggesting that these were aspects that could be more easily implemented. As was discovered in the interviews, however, these positive answers did not also mean that participants felt that the principle was being implemented in the most effective way. Concerning the use of evidence to improve future plans (principle 11), for example, participants typically referred to case study examples that can be used for inspiration. In discussions, however, participants reflected that there was little evidence being collected concerning their projects to demonstrate success. This lack of evidence-gathering resulted from uncertainty concerning the choice of indicators, who would be responsible for evidence-gathering and the expense of measuring overall impact of GI.

While participants in the survey often stated that they had been able to agree monitoring and maintenance targets and responsibilities (principle 6), maintenance emerged during the interviews as being one of the biggest challenges to the implementation and sustainability of GI. Challenges were both strategic and practical. At a strategic level, participants reported that maintenance contracts are often poorly written in terms of their applicability to GI—being focused on outputs (e.g. grass cutting) rather than outcomes (e.g. maintain habitat, increase biodiversity). Participants also expressed concern that the responsibility for ensuring correct maintenance of a development can fall to multiple actors, including public bodies (such as highways authorities as well as water utilities), councils and private developers—all of whom have separate remits and many of whom “won’t really give two hoots about the amenity value or implications of the design” (PN2). At a practical level, these strategic problems are then compounded by maintenance being conducted by companies without sufficient skills or funds to sustainably manage GI.

As can be seen from Fig. 2, it is not straightforward for our participants to implement these social principles—there are individuals finding it difficult to implement every principle and, if one agrees that the principles are important, one would hope that all participants could answer ‘able’ or above. The principles for which more participants selected ‘not able’ or ‘only somewhat able’ are 2, 3, 5, 9 and 13. These are principles that require national or strategic policy (funding, audits, standards) and/or require explicit attention to politics of identity or distributive justice (trade-offs, public interest, inclusion policies). The challenges that participants face concerning these principles for the most part pertain to the planning process in the UK and require significant political engagement, rather than technical improvements to GI. Many participants were frustrated that GI elements of projects are frequently considered to be the last, often optional add-ons, rather than essential targets. Concerning new housing developments, especially, GI often “falls into the same category of things like play areas for children […] which is disappointing” (PN3).

Participants also expressed concern regarding power asymmetries between developers and planning authorities, which frequently results in diverging from initial plans. One participant commented:

[…] even if you have quite a good vision of what green infrastructure you want, it gets value-engineered out during the process of delivery because it’s seen as ‘nice to have’ rather than a fundamental aspect of a good place, a successful place. (PN11)

As a result of being ‘nice to have’, participants argued that GI often becomes value-engineered out of projects by developers that exploit the power asymmetry that exists between developers and planning authorities. This power imbalance was predominantly seen as stemming from weak national policies concerning the general planning system combined with risk-averse, austerity-hit planning departments lacking the skills and resources to design and enforce strategic development plans (where GI is often an add-on to peoples’ roles). As a result, the quality of GI in new housing developments, especially, often becomes tied to the value of land, with councils in areas of low land value often pressured into accepting planning applications that do not fit their strategic plans in terms of GI in order to meet housing supply. This unequal access to GI is compounded by developers frequently resisting delivering 10% affordable housing *as well as* high-quality GI as was noted by one of our participants:

When there’s a requirement for 10% affordable [housing] and high-quality green infrastructure, the developer starts to get […] resistant, uncomfortable, they’ll start to suggest they’re being asked for too much. (PN13)

Such resistance on the part of developers in large part stems from the view that GI will either be too expensive or will be too complicated to implement, especially where local planning guidance is unclear and regulatory barriers will impede swift progression. In the face of these challenges, however, participants frequently spoke positively of the effect of the relatively new Building with Nature (BwN) standards—an accreditation scheme that defines high-quality GI at each stage of the development process and can be used by planning authorities to set out specific local-level expectations in their development plans and could be used as one element of ‘best value’ in public procurement.

### Practitioner views concerning stages

**Fig. 3:**
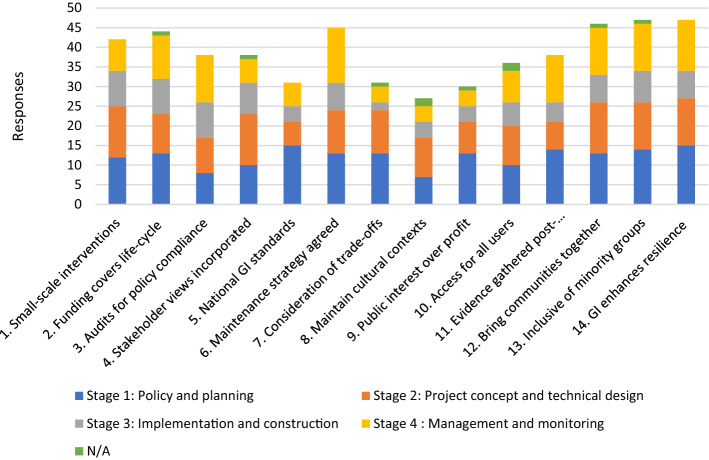
The stages at which participants think social principles should be applied. Participants were given the option of selecting multiple stages, they could also select ‘not applicable’ if the principle did not apply to their work

Figure 3 illustrates the overall findings concerning the stage(s) of a GI project at which participants felt the principles should be applied. These stages are: (1) policy and planning; (2) project concept and technical design; (3) implementation and construction; and (4) long-term management and monitoring. In general, participants felt the principles should be applied to at least one stage for the GI project cycle, with very few participants selecting ’not applicable’ (these responses accounted for less than 10% for each principle). The most notable principle where more than one person selected this option was principle 8 (in keeping with cultural context) and principle 10 (access all year). Except for participant PN11, who selected this option for three principles, there was no pattern to the ‘not applicable’ choices.

Unsurprisingly, given that principles tend to relate to strategic and policy positions, stage one (policy and planning) was selected by the most participants regarding 10 of the 14 principles; and overall stage one was the most selected stage by participants across all principles. Only principle 8 was selected for this phase by less than half of our participants. This result can be explained by the fact that participants generally highlighted the need for principle 8 to be context-dependent and should, therefore, be applied on a case-by-case basis.

Stage two (design) was often important when stage one (policy and planning) was important, occasionally becoming the stage when the principle was the most important (principle 1, 4, 8 and 12). Stage two was also the second most selected stage by participants. Stage four (monitoring and maintenance), often neglected in the literature on GI, was also seen as important for many of the principles, with more than half of our participants selecting this stage in seven of the principles. This fourth stage was selected as the most important stage for principles 3 and 6, which relate to audit and monitoring. Conversely, principles 5, 7, 8 and 9 were not selected as important by many participants for this stage, but these are strategic issues to be addressed by policy and cannot be addressed once a project has been completed. One might have expected more participants to select the fourth stage for principles 5 and 10 which suggest the need for monitoring and for appropriate maintenance accordingly.

Therefore, it is the beginning and the end of the project cycle that seem to be most important, with the majority selecting stages one, two or four for each principle. Conversely, whilst some participants did select the third phase (implementation and construction), this was nearly always selected by fewest participants for each principle. For some principles, this is relatively easy to understand—for example principle 7 (concerning socio-economic trade-offs) needs to be considered during policy and design and cannot be addressed by construction alone. In other cases, stage three (implementation and construction) was selected by over half the participants, so many participants did think the principles (particularly principles 1, 2 and 3) should be considered, but other stages were selected more frequently. Given the importance of engaging developers in the previous design stage of projects, practitioners may have felt it less important to set out the need for the application of principles to continue into the construction stage. Where stage three was selected, this was frequently done with the aim of demonstrating the need for the principles to be applied early and continuously:

You get people involved from the earlier stages and then there’s obviously clear milestones through each of those stages where a scheme, or a plan, or a design is going through to actually get built out. Checking and keeping people onboard is really important so that’s why I said it applies in all [stages]. (PN8)

Overall, our results suggest that the social principles are more important at strategic stages and help draw attention to the often-neglected maintenance part of GI projects. Moreover, there is a recognition amongst practitioners of the need to consider the practicalities of maintenance and the challenges faced by those undertaking maintenance at the strategic phase of the process. As one participant made clear, this requires early involvement of those tasked with maintaining GI:

[The projects I work on receive] capital funding, they don’t come with revenue [funding], so anything we’re creating has a maintenance cost […] So, as much as possible […] we try and get our colleagues who are responsible for maintenance involved in the design and […] that can often shape what those features are put in. (PN10)

## Discussion

Our findings indicated that practitioners in the UK are in favour of incorporating social principles into the concept of GI that reach beyond the provision of socio-economic benefits. Results also showed that practitioners are encountering numerous challenges in applying the social principles suggested in this paper. In terms of agreeing on the social principles themselves, GI professionals were most in favour of principles that ensured the inclusivity, equal access to and long-term funding options for GI, confirming our literature review findings. Where participants were less aligned, both between themselves and with the literature, concerned principles that seek to ensure that local cultural contexts are maintained, and that stakeholder preferences are prioritised when implementing GI. These principles are often seen as key measures to avoid possible gentrifying effects of GI and other greening initiatives, as Curran and Hamilton (2012) and Wolch et al. (2014) have in concerning greening strategies in the United States and China respectively. These findings are therefore relevant beyond the context of the UK. While participants were sympathetic to these issues and often shared such concerns, they also felt that the principles as suggested here were too generalised and could produce harmful social consequences in turn. GI, it was felt, is not a panacea to solve the problems of the planning system—yet these Green Infrastructure principles have the potential to form a more collaborative and future-oriented framework through which to work.

In conceptualising GI as a positive framework and attending to the processes as well as outcomes, the question of the applicability of social principles can be shifted to one of social practices. In other words, instead of attempting to pin down another definition of GI, researchers and policymakers should agree on processes through which GI is best achieved. In setting out social good practice approaches, practitioners would therefore be in a better position to resist both the depoliticisation of GI (Finewood et al. 2019), ensure GI is socially inclusive (Haase et al. 2017) as well as maintain the malleability of the GI concept to fit local requirements and geographies (Lindholm 2017). Our findings also raise questions concerning the spatial scale at which such good practice approaches should be applied (e.g. local, municipal, regional) and the extent to which these approaches should differ depending on the spatial scale. These issues concerning scale should be explored in further research.

Our results showed that professionals involved in GI are encountering numerous challenges in applying the principles suggested in this paper. In part these challenges stem from GI still being predominantly seen as a ‘nice to have’ addition, rather than the starting point of an integrated planning approach. Other challenges included the differing aims of the actors involved in maintaining GI and the perceived power asymmetry between developers and planning authorities. These findings have important policy implications, helping to inform national legislation regarding incorporating GI into the planning systems, and the resulting local GI strategies—many of which are currently out for consultation. As our results demonstrate, however, applying such a localised approach successfully will be a significant challenge given the ongoing effects of austerity cuts on councils (Gray and Barford 2018) as well as the current housing crisis (Gallent et al. 2018). Participants viewed the need for housing as being leveraged against planning authorities to gain planning permission by land owners and developers while minimising GI commitments. Clearly GI does not exist outside of the socio-political spaces in which it is implemented, yet more work is needed to understand how these socio-political contexts influence the evolution of GI through changes to its financing, implementation and governance in both the UK and other national contexts (see Mell 2018). In this respect it is important to note that participants found the Building with Nature accreditation scheme useful to centre GI as an integral element of the process as well as define local-level expectations for planning authorities’ development plans.

Our results also indicate that the timing of when social principles become applied in the process of GI implementation is central to ensuring their outcome. Early incorporation at strategic stages is seen as key in terms of bringing developers on board and ensuring compatibility with the specifications of other actors (such as utilities providers) and councils (or others) involved in the maintenance of GI. While the challenge of maintenance, which is key for ensuring the continued sustainability and place-keeping of new developments (Buijs et al. 2016), is not an issue that is unique to GI (see Schoonnees et al. 2019), it is nonetheless a challenge that requires systemic resolution. Where participants reported some success in terms of applying the social principles put forward in this paper, this success was predominantly predicated on pulling together multiple actors, creating iterative design processes and agreeing maintenance *outcomes* from the outset. Such a sophisticated approach again requires GI to be considered the guiding framework for developments, rather than a ‘nice to have’ add-on.

The findings presented here concerning GI can, as Nesshöver et al. (2017) have suggested, usefully assist nature-based solutions (NBS) in understanding the challenges it will face (Kabisch et al. 2016). The need to incorporate multiple actors in the design phase of sustainable projects and consider maintenance from the outset, as outlined in this paper, speaks directly to current debates concerning NBS, where issues concerning the costs and design of maintenance are increasingly becoming key considerations (Emilsson and Ode Sang 2017; Keesstra et al. 2018). NBS research should also take note that, although NBS might become foregrounded in national policy as requiring integrated and systemic approaches (Nesshöver et al. 2017), the lessons from this research indicate that such policy efforts might not be reproduced in practice. More work is needed to understand the dynamics and local politics that take place throughout development processes, how these affect social outcomes of GI and whether they influence current conceptual frameworks of GI. Although our data referred mostly to urban or local-scale GI, this is an issue which will also be particularly relevant to GI planning at the landscape scale given the increased number of stakeholders and actors involved. Future research should be aimed at assisting the design of planning GI guidelines and testing their applicability with planning authorities. Through focusing on project stages and actors involved in implementing social principles, this paper has furthered our understanding of these local-level dynamics. Our study could be augmented through expanding the sample size, which would allow for a more in-depth understanding of the geographically specific challenges that GI professionals face. Augmenting sample size in this way would also incorporate the views of participants who are currently less engaged in GI, as this might also shed light on another set of challenges. Our mixed method approach highlighted the methodological difficulties of defining GI, the spatial scales at which it is applied, whether GI is on private or public land, and whether or not principles (social and otherwise) are being applied in the most effective way. We therefore recommend that further research into GI continues to utilise qualitative methods to fully explore practitioners’ experiences concerning the implementation of GI.

## Conclusion

While much of the existing literature focuses on the ecological and socio-economic benefits of GI, as one form of NBS, this paper questions how these benefits can be experienced in a more socially inclusive manner. We approached this issue through presenting practitioners engaged with GI in the UK with 14 social principles which we selected from the literature. We asked participants to what extent they believed the principles applied to GI, to what extent they felt able to apply them in their work, and when in the project cycle they thought each principle should be applied. In attending to the project cycle, we were able to draw out the importance of the myriad of actors that become involved in GI projects and the roles and influences they have on the planning and implementation processes. Through this focus on the politics that surrounds how GI becomes both designed and maintained, we have drawn attention to the oft-overlooked maintenance phase of GI—highlighting the need for decision-makers to recognise that the complexity of maintenance exceeds the issue of cost. Policymakers could require public bodies (such as highway authorities as well as water utilities) to have both the expertise and funds to constructively engage in the design and maintenance of GI for benefits that exceed technical engineering aims. Cross-silo thinking is therefore needed not just to initiate GI projects but to aspire towards positive social outcomes as well as ensuring long-term success. Setting out social good practices will benefit in this regard. Maintenance plans should be set out at the initial planning application stage, this will also reduce the possibility for GI to be ‘engineered out’ of projects during implementation. Further work is needed to explore how the responsibilities for the management of GI can be simplified to ensure common maintenance outcomes (e.g. maintain habitat, increase biodiversity) rather than outputs (e.g. cut grass).

In focusing on the politics of implementing GI, we have also sought to explicitly link the issue of procedural aspects of GI to the current context of housing policy in the UK. In doing so, we have argued that the effects of austerity and the housing crisis act as significant obstacles to ensuring that the benefits of GI can be enjoyed equally. The hollowing out of the state in the UK likely means that GI projects will need to become more creative to gain long-term funding (Mell 2016). To overcome these challenges and assist in this process, we see value in setting out good practice social approaches to GI that can be adopted by local planning authorities in order to strengthen their respective strategic development plans. To avoid being watered down and employed solely as a technical solution, however, GI, and NBS more widely, need to be politicised in the planning and procurement processes. As Lindholm (2017) has argued, this politicisation means to discuss and flesh out the ‘why’ and the ‘where’ questions concerning GI, before more technical questions should be considered. The social principles suggested here provide a starting point for this politicisation such that GI might be better governed and more-inclusively designed in the future. Given the resonances with literature concerning the social challenges involved in greening and NBS strategies in other national contexts, these findings have wider relevance beyond the UK.

## Electronic supplementary material

Below is the link to the electronic supplementary material.

Supplementary material 1 (PDF 719 kb)

## Footnotes

1 Conference attendance lists gave names and affiliations only. Researchers only contacted those who’s email addresses could be found through publicly available means.
