# Supplementary material for: “It’s on the ‘nice to have’ pile”: Potential principles to improve the implementation of socially inclusive Green Infrastructure
Source: Ambio. 2020 Sep 30;50(8):1574–86. doi: 10.1007/s13280-020-01372-2 (PMC7525230; doi:10.1007/s13280-020-01372-2)
Supplement: Supplementary file 1 — Supplementary material 1 (PDF 719 kb) [file 13280_2020_1372_MOESM1_ESM.pdf]

Ambio

Electronic Supplementary Material

This supplementary material has not been peer reviewed.

Title: “It’s on the ‘nice to have’ pile”: Potential principles to improve the implementation of socially inclusive Green Infrastructure

Authors: Dan Fisher, Kirsty Blackstock, Katherine Irvine

Q1.

Welcome! The goal of this survey is to investigate understandings of Green Infrastructure amongst practitioners in the United Kingdom. The survey is being conducted by [redacted]

The survey has three short sections. The main focus of the survey is a list of 14 social principles that could be attributed to Green Infrastructure. We will mainly ask you questions based around these 14 principles – there are no correct answers, instead we are interested in hearing your opinion. We expect that completing the survey should take no more than fifteen minutes.

Your participation in this survey is voluntary and you are not obligated to complete this questionnaire. You may ask for your contributions to be withdrawn from the survey at any time before the 1st of March 2020, by emailing [redacted]

Q2.

Section 1. In this section we will ask you some questions about your current employment and what you understand the term Green Infrastructure to mean.

Q3.

1.1 Which of the following best describes your current professional setting or sector? Please select the most relevant option.

- ○ Environmental / Conservation NGO
- ○ Private sector: environmental consultancy
- ○ Private sector: other (please specify) ______
- ○ National government
- ○ Local government
- ○ Public sector: environmental agency or department
- ○ Public sector: other (please specify) ______
- ○ Academia
- ○ Other ______

Q4.

1.2 Please name the organization that you work for

______

Q5.

1.3 What is your job title in that organisation?

______

Q6.

1.4. In your own words, how would you define the concept of Green Infrastructure? We realise that this is not an easy question, but we are interested in *your* understanding and use of the concept, so please to not use Google to check for a 'correct' definition!

Q7.

Section 2. In this section we are interested in which social principles you would attribute to the concept of Green Infrastructure.

Q8.

Below you will see a list of principles that have been attributed to the concept of Green Infrastructure in reports and academic work. **We would like to know which of these principles *you* think should be applied to Green Infrastructure. For each principle, please select to what extent you agree or disagree that the principle should be applied to the concept.**

	Strongly Disagree	Disagree	Neutral	Agree	Strongly Agree	Don't Know
Green Infrastructure should include small-scale interventions that evenly distribute access to nature for all residents.	○	○	●	○	○	○
Funding for Green Infrastructure should cover the full life-cycle of projects (i.e. including the maintenance and monitoring costs).	○	○	●	○	○	○
There should be regular checks or audits in place to ensure that Green Infrastructure projects comply with relevant policies and procedures.	○	○	●	○	○	○
The preferences of residents and stakeholder groups should be incorporated into Green infrastructure projects, even if these limit other goals.	○	○	●	○	○	○
There should be national Green Infrastructure standards that are embedded within planing and social policy.	○	○	●	○	○	○
	Strongly Disagree	Disagree	Neutral	Agree	Strongly Agree	Don't Know
There should be clear targets and responsibilities for the monitoring and maintenance of Green Infrastructure projects post-installation.	○	○	●	○	○	○
Socio-economic trade-offs associated with Green Infrastructure need to be considered, and negative impacts minimised especially in areas of high inequality.	○	○	●	○	○	○

	Strongly Disagree	Disagree	Neutral	Agree	Strongly Agree	Don't Know
Green Infrastructure should be in keeping with existing land uses and cultural contexts of an area, even if these are 'industrial'	○	○	◉	○	○	○
Private profit should not be prioritised over public interest when seeking funding from private actors for Green Infrastructure.	○	○	◉	○	○	○
Access for all users throughout the year should be included in Green Infrastructure.	○	○	◉	○	○	○
Evidence from completed projects should be used to revise Green Infrastructure goals and future projects.	○	○	◉	○	○	○
Green Infrastructure should help to bring communities together.	○	○	◉	○	○	○
Green Infrastructure projects should be inclusive of minority and disadvantaged groups, working to ensure they benefit following installation.	○	○	◉	○	○	○
Green Infrastructure should enhance community resilience (i.e. the ability of a community to use locally-available resources and withstand adverse situations such as climate change).	○	○	◉	○	○	○

Q9. Please feel free to use this text box to add a principle to our list, or to leave a comment concerning your answers.

Q10. **Section 3.** In this section we are interested in determining when a social principle should be applied during the life-cycle of a Green Infrastructure project.

Q11.
We have identified and defined four separate stages below during which our social principles for Green Infrastructure could be applied. **In the table, please select which stage(s) you think each principle should be applied at, you may select multiple stages.** If you do not think that a principle should be applied or it is not relevant to your work, please select 'N/A' (Not Applicable).

Stage 1: Policy and strategy – Refers to government policy-setting concerning Green Infrastructure, as well as steering of the GI concept by e.g. built environment organisations and consultancies.

Stage 2: Project concept and technical design – Refers to the design and planning stage of specific GI projects that will be applied on a local scale by e.g. planners, architects, project management services in collaboration with local government.

Stage 3: Implementation and construction – Is the stage during which plans are put into practice, often by developers and contractors that have not been involved in stages 1 and 2.

Stage 4: Long-term management and monitoring – Concerns the post-construction phase during which time GI needs to be maintained and used.

	Stage 1: Policy and strategy	Stage 2: Project concept and technical design	Stage 3: Implementation and construction	Stage 4: Long-term managament and monitoring	N/A
Green Infrastructure should include small-scale interventions that evenly distribute access to nature to all residents.	☐	☐	☐	☐	☐
Funding for Green Infrastructure should cover the full life-cycle of projects (i.e. including maintenance and monitoring costs).	☐	☐	☐	☐	☐
There should be regular checks and audits in place to ensure that Green Infrastructure projects comply with relevant policies and procedures.	☐	☐	☐	☐	☐
The preferences of residents and stakeholder groups should be incorporated into Green Infrastructure projects, even if these limit other goals.	☐	☐	☐	☐	☐
There should be nationally-agreed Green Infrastructure standards that are embedded within planning and social policy.	☐	☐	☐	☐	☐
	Stage 1: Policy and strategy	Stage 2: Project concept and technical design	Stage 3: Implementation and construction	Stage 4: Long-term managament and monitoring	N/A
There should be clear targets and responsibilities for the monitoring and maintenance of Green Infrastructure projects post-installation.	☐	☐	☐	☐	☐
Socio-economic trade-offs associated with Green Infrastructure should be considered, and negative impacts minimised especially in areas of high inequality.	☐	☐	☐	☐	☐
Green Infrastructure should be in keeping with existing land uses and cultural contexts of an area, even if these are 'industrial'	☐	☐	☐	☐	☐
Private profit should not be prioritised over public interest when seeking funding from private sector actors for Green Infrastructure.	☐	☐	☐	☐	☐
Access for all users throughout the year should be included in Green Infrastructure.	☐	☐	☐	☐	☐
	Stage 1: Policy and strategy	Stage 2: Project concept and technical design	Stage 3: Implementation and construction	Stage 4: Long-term managament and monitoring	N/A
Evidence from completed projects should be used to revise Green Infrastructure goals and strategies for future projects.	☐	☐	☐	☐	☐
Green Infrastructure should help bring communities together.	☐	☐	☐	☐	☐

Green Infrastructure projects should be inclusive of minority and disadvantaged groups, working to ensure they benefit following installation.	☐	☐	☐	☐	☐
Green Infrastructure should enhance community resilience (i.e. the ability of a community to use locally-available resources and withstand adverse situations such as climate change).	☐	☐	☐	☐	☐

Q12. Please feel free to use this text box to leave a comment concerning your answers or the concept of a project life-cycle.

Q13. **Section 4.** In this section we are interested in your experiences of applying these principles in the context of your work.

Q14. Below you will see the same list of potential principles. However, in this case **we would like to know whether you have felt *able to apply* these principles in your work in the recent past (i.e. within the last 3-5 years). Please tick an option between 1 to 5, where 1 is if you *have not felt able* at all to apply the principle, and 5 if you feel you *have been able* to apply the principle with little or no difficulty.** If the principle cannot be applied to the work that you do, please tick 'N/A' (Not Applicable)

	1 (Not able at all)	2	3	4	5 (No difficulty)	N/A
Green Infrastructure should include small-scale interventions that evenly distribute access to nature for all residents.	○	○	○	○	○	○
Funding for Green Infrastructure should cover the full life-cycle of projects (i.e. including the maintenance and monitoring costs).	○	○	○	○	○	○
There should be regular checks or audits in place to ensure that Green Infrastructure projects comply with relevant policies and procedures.	○	○	○	○	○	○
The preferences of residents and stakeholder groups should be incorporated into Green infrastructure projects, even if these limit other goals.	○	○	○	○	○	○
There should be national Green Infrastructure standards that are embedded within planing and social policy.	○	○	○	○	○	○
	1 (Not able at all)	2	3	4	5 (No difficulty)	N/A
There should be clear targets and responsibilities for the monitoring and maintenance of Green Infrastructure projects post-installation.	○	○	○	○	○	○

Socio-economic trade-offs associated with Green Infrastructure need to be considered, and negative impacts minimised especially in areas of high inequality.						
Green Infrastructure should be in keeping with existing land uses and cultural contexts of an area, even if these are 'industrial'						
Private profit should not be prioritised over public interest when seeking funding from private actors for Green Infrastructure.						
Access for all users throughout the year should be included in Green Infrastructure.						
	1 (Not able at all)	2	3	4	5 (No difficulty)	N/A
Evidence from completed projects should be used to revise Green Infrastructure goals and future projects.						
Green Infrastructure should help to bring communities together.						
Green Infrastructure projects should be inclusive of minority and disadvantaged groups, working to ensure they benefit following installation.						
Green Infrastructure should enhance community resilience (i.e. the ability of a community to use locally-available resources and withstand adverse situations such as climate change).						

Q15. Please feel free to use this text box to leave a comment concerning your answers concerning your ability to apply the previous principles.

Q16. **This is the end of the survey. Thank you very much for your time.** We will send you a copy of your survey answers before the interview.

[redacted]

[redacted]
